# The Health Effects of 14 Weeks of Physical Activity in a Real-Life Setting for Adults with Intellectual Disabilities

**DOI:** 10.1155/2022/6817318

**Published:** 2022-12-01

**Authors:** Laurits Munk Højberg, Eva Wulff Helge, Jessica Pingel, Jacob Wienecke

**Affiliations:** ^1^Department of Nutrition, Exercise and Sports, Faculty of Science, University of Copenhagen, Copenhagen, Denmark; ^2^Department of Neuroscience, Faculty of Health and Medical Sciences, University of Copenhagen, Copenhagen, Denmark; ^3^Department of Sport and Social Sciences, Norwegian School of Sport Science, Oslo, Norway

## Abstract

**Background:**

The life expectancy of individuals with intellectual disabilities (ID) is reduced compared to the general population, and one of the main contributors to earlier death is inactivity.

**Aim:**

To investigate how 14 weeks of physical activity (PA) in a real-life setting affects cardiovascular fitness, body composition and bone health of adults with ID.

**Methods:**

Adults with ID were recruited into a PA-group (*N* = 52) or a control group (CON, *N* = 14). The PA-group participated in 14 weeks of PA, and body composition, cardiovascular fitness and bone health were assessed before and after the intervention. *Outcomes and Results*. Cardiovascular fitness and body composition improved from pre to post within the PA-group: Heart rates (HR) during the last 30 seconds of two increments of a treadmill test, were reduced (3.2 km/h: −4.4 bpm, *p* < 0.05; 4.8 km/h: −7.5 bpm, *p* < 0.001) and fat mass was reduced (−1.02 kg, *p* < 0.05). A between-group difference in favour of the PA-group, were observed in whole body bone mineral density (BMD) (0.024 g/cm^2^, *p* < 0.05). *Conclusions and Implications*. Fourteen weeks of PA performed in a real-life setting increased cardiovascular fitness, reduced fat mass and improved BMD in the weight-bearing skeleton in the PA-group. Increased and regular PA seems to be a promising tool to promote physical health in adults with ID.

## 1. Background

Individuals with intellectual disabilities (ID) are subjected to increased mortality, in Denmark, individuals with ID die 14.5 years earlier than the general population [1]. The increased mortality is in part attributed to decreased physical health, high levels of inactivity and obesity which can lead to lifestyle diseases such as type II diabetes, cardiovascular disease and poor bone health [2–4]. Physical activity (PA) has been suggested as an effective way to improve the health in individuals with ID [2, 5]. Previous investigations of individuals with ID have shown that the group respond to physical activity similarly to the general population. Evidence from controlled trials and systematic reviews show improvements in cardiovascular fitness following 80–135 minutes of aerobic training per week for 8–16 weeks [6, 7]. The evidence regarding resistance training indicates that 8–24 weeks of resistance training increases muscle strength of persons with ID [8, 9]. Interventions with both aerobic and resistance training has shown that the participants experienced the benefits from both these modalities, within 10–20 weeks [10–12]. PA for individuals with ID seem to induce reductions in body weight and fat mass [12], and improved bone health [13], but the results are equivocal within these areas, and more research is warranted. Improvements in the above-mentioned domains have a positive influence on general health. Improved cardiovascular fitness is associated with reduced risk of early death from cardiovascular and respiratory diseases [14], and increased muscle strength in the lower body can prevent falls and fractures [15]. The mentioned studies have generally used supervised exercise interventions, with researchers controlling the PA performed by the participants. The evidence from these studies is fundamental, but the translation into real-life, health-promoting exercise programs for the population with ID is difficult and demand further research [2, 5]. Hence, the aim of the present study was to investigate how 14 weeks of varied and adapted PA performed in a real-life setting, 5 days per week affects the health of adults with ID. To fulfil this aim, we collaborated with a local daily activity centre, where adults with ID are enrolled for a year and participating in PA at the centre 5 days a week. Thus, an already feasible intervention could be investigated on a large number of participants. We hypothesised that a PA-group would improve their health from pre-to post-test compared with a control group. In addition, we hypothesized that a group of participants, who had already attended the activity centre at least one school year prior to the study (2^nd^-year participants), would have a better health profile at PRE-tests than the participants, who started their first year at the centre when entering the study (1^st^-year participants), due to impact of the PA that the 2^nd^-year participants had already performed. For the same reason, we expected to find the largest improvements in the selected health parameters in the 1^st^-year group over the course of the study.

## 2. Methods

### 2.1. Intervention and Setting

The intervention consisted of varied forms of PA, performed in four periods of 3-4 weeks in duration, for a total of 14 weeks. Period 1: Dance, cycling, basic ballgames, outdoor fitness, and athletics; Period 2: Adventure, motor training, football (soccer) and maritime outdoor activities. Period 3: Body theatre, swimming, floorball, and e-sport. Period 4: Body theatre, racket sports, volleyball, and fitness. The activities were performed at the activity centre; the Sports School for Adults with Developmental Disabilities (SSADD) in Copenhagen, Denmark. Adults with ID attending the SSADD, referred to as “participants,” were recruited into the PA-group. Each participant chose one of the above activities, in each period. Five days per week, they had 150 minutes of scheduled PA, in two blocks of 75 minutes with 45 minutes of break time in between. The participants in the PA-group were served snacks in the form of ryebread, fruits, and vegetables and a healthy lunch while at SSADD. The SSADD has a capacity of ∼50 adults with different grades of ID, per school year. Each year approximately half of the participants continue at SSADD for an additional year (2^nd^-year participants), and ∼25 new participants start at the school (1^st^-year participants). The personnel at SSADD aims at improving the physical, social, and personal competences of the individuals attending the institution, through the use of inclusive PA and sports, and the activities are adjusted to meet the capabilities of the individuals attending the SSADD.

### 2.2. Participants

**Figure 1:**
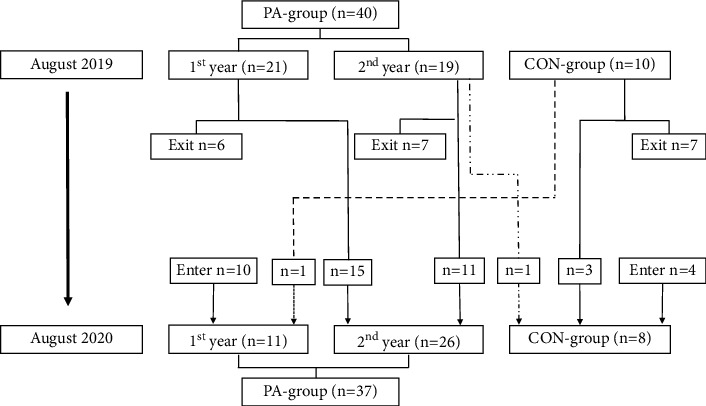
Flow chart of the movements between the groups of the participants between the two rounds of experiments, from August 2019 to August 2020.

Fifty-two adults with ID (26.7 ± 1.2 years) were recruited from the SSADD (PA-group). The PA-group was further divided into a 1^st^-year group and a 2^nd^-year group. A control group (CON-group) of 14 adults (29.1 ± 1.7 years) were recruited from other institutions and activity centres in Copenhagen. The institutions, where the participants in the CON-group were recruited from, did not have the same focus on physical activity as the SSADD. Instead, they had a focus on activating the participants creatively and socially through smaller group activities (e.g., organizing cultural events, exhibitions, and media production), but also adapted ball games and morning gymnastics on a smaller scale. Some participants in the CON-group were active on an organized floorball team, which met for practice twice a week and played matches occasionally. The participants in the CON-group were asked to live their lives as usual. This study was carried out with approval of The Danish Committee on Health and Research Ethics (H-19017828). All participants were thoroughly informed about the study (one-to-one) by a member of the research team, where they were made aware of their rights as participants and were able to ask questions before giving their written consent to participate in the present study. Family members or guardians were present when needed to support the conversation. Inclusion criteria were presence of ID and an age of 18–68 years, while being under legal guardianship was an exclusion criterion. Thus, only participants who were able to decide for themselves if they wanted to participate or not were included. The study followed with the Helsinki declaration. Since the project ran for two years, some participants were tested PRE and POST twice, with a year between the intervention periods. Between the two rounds of measurements, the participants were allowed to change groups; for example, many participants the 1^st^-year group in the first round continued at SSADD and were then included in the project in the 2^nd^-year group (Figure 1). Some participants who had initially given their consent to participate in the study chose to withdraw their consent, during the course of the study. These circumstances resulted in different numbers of participants both between the different tests and between the PRE and POST measurements. An overview of the number of measurements performed in the different tests can be found in Appendix 1. The grade of ID of the participants varied from mild to moderate and consisted of individuals diagnosed with Down syndrome, autism spectrum disorders, cerebral palsy, and few participants with Fragile X syndrome, William syndrome, tuberous sclerosis, Bardet Beidel syndrome, and Noonan syndrome. Some participants had additional diagnoses such as epilepsy or attention-deficit hyperactivity disorder (ADHD).

### 2.3. Testing Procedure

The individuals in the PA-group were recruited during their first week at SSADD over two intervention rounds, in 2019 and 2020. After recruitment, the participants in the PA-group underwent the tests described below over the course of two weeks. Participants in the CON-group were recruited during this period and were tested after the PA-group. From PRE-to POST-tests, 14 weeks passed on average in both groups. All tests were carried out at the facilities of the Department of Nutrition Exercise and Sports, University of Copenhagen.

### 2.4. Assessment of Physical Activity

To measure the total amount of daily PA performed by the participants, accelerometry using the Axivity AX3 system were performed. Accelerometers (Axivity, Newcastle UK) were attached to the right thigh of the participants for periods of seven days, using medical tape and waterproof artificial skin (Opsite Flexifix®). The accelerometers were checked daily in the weekdays to ensure correct positioning. If the accelerometer had detached it was reattached and recalibrated. Based on the threshold values of standard deviation of acceleration and the derived deviation [16], the software ACTi4 (ACTI Corp., Copenhagen, Denmark) was used to discriminate the accelerometer data into different PA types (sitting, standing, walking, walking on stairs, running, cycling and total number of steps). This enabled the measurement of steps taken pr. Hour and minutes of activity pr. Week. The participants were asked to register their sleep schedules (hours slept) with the help of family or personnel at their home. Forty-three participants were divided between six different activities: football (*n* = 9), motor training (*n* = 5), fitness activities (*n* = 9), outdoor activities (*n* = 13) and cycling (*n* = 7). Not all participants were able to wear the accelerometer for seven days. In these cases, the days they had completed, were included in the data.

### 2.5. Dual X-Ray Absorptiometry (DXA) Scans

The outcome measures of interest to the present study were body composition measures (e.g., fat mass (kg) and fat free mass (kg)) and areal bone mineral density (BMD, g/cm^2^). The DXA-protocol consisted of a whole-body scan [17], and scans of the lumbar vertebrae (L1–L4) and bilateral proximal femur, in that order (Lunar iDXA, GE Healthcare). DXA scans of the lumbar and femur regions are routinely used as clinical tools for diagnosing osteoporosis [18]. The participants' weight and height were recorded prior to the scans (Soehnle Professional Scale, Soehnle, Germany). The participants were asked to remove all metal objects (jewellery, buttons etc.) from their body and clothing prior to the scans. The participants were asked to arrive to the tests in a fasting state. Not all participants were able to meet this request, so their fasting state was recorded at the PRE-tests (fasting/non-fasting). The participants were asked to arrive to the POST-test in the same fasting state as at the PRE-test. The participants were placed in the middle of the scanning bed with their arms held to the sides of the body, their hands in neutral position and their palms on the side of their thighs. With the knees bent in a 90 degrees angle, a foam block was placed under the participant's calves for the lumbar scans to ensure that the lower back was placed correctly on the scanning bed. During the femur scans, the participants were asked to rotate their legs inwards from the hips, while their feet were strapped to a plastic trapezoid. This was done to ensure the right position of the femur neck, and to promote reproducibility of the measurements.

### 2.6. Oxygen Uptake Protocol and Heart Rate

The submaximal oxygen uptake (VO_2_)-protocol of the present study was inspired by study by Ebbeling and colleagues [19], where a submaximal treadmill test for estimation of VO_2_ max was developed. The test consisted of a 4-minute warm up with 0% incline at two different speeds, two minutes at 3.2 km/h followed by two minutes at 4.8 km/h. The warm-up was followed by 1 minute at standing rest. After the rest the participants performed the test, a bout of 8 minutes with two intensities. First, the participants walked at 3.2 km/h and 5% incline for 4 minutes, immediately followed by 4 minutes at 4.8 km/h and 5% incline. Online breath-by breath analysis was performed during the exercise protocol using a metabolic cart (Masterscreen CPX, CareFusion). Participants wore a mask with ∼70 ml dead space, which was directly connected to the gas analyser and flow sensor. The system was calibrated before each trial. The heart rates (HR) were sampled using POLAR H3 heart rate sensors. An experimenter noted the heart rates of the participants every 10 seconds during the exercise protocol. The area under the curve (AUC) of the VO_2_-curves during intensity transitions is an estimate of oxygen uptake kinetics. The first 20 seconds of the transitions were omitted from the analyses to exclude phase 1 kinetics and investigate phase 2 kinetics. AUC were then calculated for the subsequent 100 s. An increase in AUC of the VO_2_-curve during similar intensity transitions and a fixed time frame, is a measure of increased total oxygen consumption.

### 2.7. Data Analysis and Statistics

Data were analysed using R statistics. Linear mixed models (LLM's) were used to analyse changes within and between groups (“lme4” R-package [20]). In the first LLM, the group variable had two levels: PA and CON. In the second LLM, the group variable had three levels: 1^st^-year group, 2^nd^-year group and CON. The LLM's included the age, gender, and diagnosis of the participants as well as the test round (i.e., 2019 and 2020), as fixed effects, and the participant IDs as a random effect. LLM's were chosen, for their ability to handle missing data. The model applied in the present study in *R* terminology is shown below:

Post hoc analysis of the models was used to investigate the changes from PRE-to POST-tests between and within the groups (“multcomp” R-package [21]). The *p*-values were corrected for multiple comparisons with the single step method. The critical value for statistical significance were assumed at an alpha level *p* ≤ 0.05. Unless otherwise stated, data are presented as mean ± standard error (SE). Percent changes within groups are calculated as the change from PRE-to POST-test, and percentwise differences between groups are calculated as the change score between the PA groups and the CON group, relative to the CON-group PRE-test value. Model estimated means and 95% confidence intervals [Lower Level, Upper Level] are reported for selected body composition, bone health and cardiovascular measures. To compare the variability of the different measures included in the study, and to gain insight about validity of our data, we calculated coefficients (CV = model SD/model mean) of variation for selected outcomes at the PRE and POST measurements.

## 3. Results

### 3.1. Demographic Data

The demographic data collected at the PRE-measurements is presented in Table 1. Some participants were tested in both 2019 and 2020. They have contributed to the demographic data once, in 2019. An overview of the total number of data points included in the measurements, can be found in Appendix 1.

### 3.2. Daily Physical Activity

**Figure 2:**
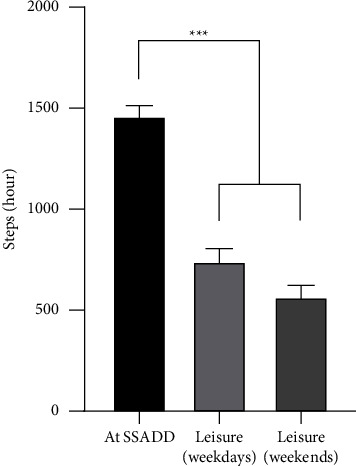
The number of steps taken pr. Hour while the participants were at SSADD, during leisure on weekdays after the school day ends and during weekends. Measurements were made over a 7-day period. Error bars: Standard error of the mean. Significance codes: “^*∗∗∗*^” denotes *p* < 0.001, “^*∗∗*^” denotes *p* < 0.01, “^*∗*^” denotes denotes *p* < 0.05.

During one week, the participants in the PA-group on average took more steps/hour (*p* < 0.001) while at SSADD (1453 ± 60 steps/h) compared to leisure hours, both on weekdays (738 ± 67 steps/h) and in the weekends (564 ± 58 steps/h) (Figure 2). The PA-group performed on average 407 minutes of PA/week during the total of 1500 minutes they spent at the school. This included activities other than standing, sitting, or lying down. As mentioned, they had 150 minutes of scheduled PA/day (equivalent of 750 minutes/week).

### 3.3. PRE-Test Measurements

**Figure 3:**
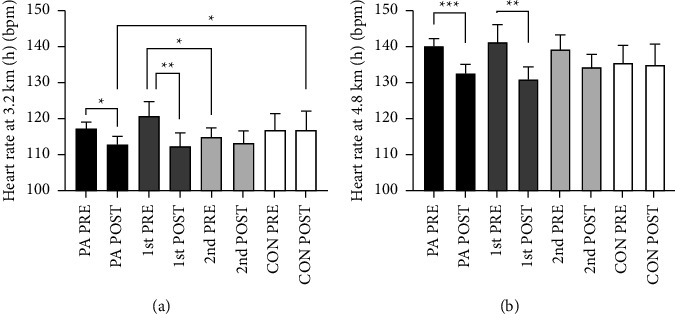
The average heart rates during the last 30 s at 3.2 km/h (a) and 4.8 km/h (b) for the PA-, 1^st^-year, 2^nd^-year and CON-group. Significance codes: “^*∗∗∗*^” denotes *p* < 0.001, “^*∗∗*^” denotes *p* < 0.01, “^*∗*^” denotes denotes *p* < 0.05.

Raw PRE-test values are presented in Table 2. The comparison between the 1^st^-year group and the 2^nd^-year group showed a higher total body weight (4.7%, mean = 3057.5 g, CI [912.5 g, 5202.5 g], *p* < 0.05) and a higher fat mass (11%, mean = 2879.4, CI [1136.1 g, 4622.7 g], *p* < 0.001) in the 1^st^-year group. In addition, the 1^st^-year group had a higher android (16.2%, mean = 409.7 g, CI [179.8 g, 639.5 g], *p* < 0.001) and gynoid fat mass (8.9%, mean = 460.1, CI [123.8 g, 796.4 g], *p* < 0.05) than the 2^nd^-year group. The 1^st^-year-group had a higher heart rate (HR) compared to the 2^nd^-year group during the last 30 s at 3.2 km/h (5.0%, mean = 7.9 bpm, CI [1.6 bpm, 14.2 bpm] *p* > 0.05) (Figure 3). The PA-group had lower BMD compared to the CON-group in L1–L4 (−6.8%, mean = −0.036 g/cm^2^, CI [−0.068 g/cm^2^, −0.004 g/cm^2^] *p* > 0.05). The *T*-score distribution of the right femur neck of the PA-group was: *T* ≤ −2.5 (osteoporosis): 0%, −2.5 > *T* ≤ −1 (osteopenia): 23.5%, −1 > *T* < 0 : 29.4% and *T* ≥ 0 : 43.1%. The CON-group distribution was: *T* ≤ −2.5 (osteoporosis): 0%, −2.5 > *T* ≤ −1 (osteopenia): 28.6%, −1 > *T* < 0 : 35.7% and *T* ≥ 0 : 35.7%. As no differences were observed between the right and left sides at PRE-tests, only data from the right side is presented.

### 3.4. Pre-To Posttest Changes

#### 3.4.1. Body Composition

Table 3 presents the model estimated Δ-values (POST-PRE) within the groups, and the differences in change scores between the groups (e.g., PA-group Δ-value-CON-group Δ-value). Analyses of body composition outcomes form the whole body DXA scans revealed that the total PA-group lost −4.0% fat mass (mean = −1016.4 g, CI [−1854.7 g, −178.1 g], *p* < 0.05), with the 1^st^-year group lost −5.8% fat mass (mean = −1707.3 g, CI [−2854.5 g, −560.1 g], *p* < 0.05). For the 1^st^-year group, parts of the fat loss were located at the android and gynoid regions (−7.4%, mean = 346.1 g, CI [−570.4 g, −121.9 g], and −7.1%, mean = 199.4 g, CI [−352.6 g, −46.1 g], *p* < 0.05, respectively). However, no between group differences were observed in the body composition outcomes (all *p*-values >0.05).

#### 3.4.2. Bone Mineral Density

A within-group change was observed in the PA-group, the BMD of the right femur neck increased from PRE to POST (mean = 0.017 g/cm^2^, CI [0.004 g/cm^2^, 0.030], *p* < 0.05) (Table 3). A difference in change the score for whole body BMD (2.0%, mean = 0.024 g/cm^2^, CI [0.006 g/cm^2^, 0.042 g/cm^2^], *p* < 0.05) between the PA- and CON-group, in favor of the PA-group, was observed.

#### 3.4.3. Cardiovascular Fitness

Within-group changes was observed in the PA-group as a reduction in HR during the last 30 s at both 3.2 km/h (−3.8%, mean = −4.4 bpm, CI [−7.9 bpm, −1.0 bpm] *p* < 0.05) and 4.8 km/h (−5.4%, mean = −7.2 bpm, CI [−10.7 bpm, −3.6 bpm], *p* > 0.001) (Figure 3). This reduction in HR was more pronounced within the 1^st^-year group (3.2 km/h: −7.0%, mean = −8.5 bpm, CI [−13.8 bpm, −3.2 bpm], *p* < 0.01) and (4.8 km/h: 7.2%, mean = −10.2 bpm, CI [−15.8 bpm, −4.5 bpm], *p* > 0.001). However, no between group differences were observed in the HR measurements, and VO_2_-kinetics during the transitions of the treadmill test did not change (*p* > 0.05).

### 3.5. Post-Test Differences

Between-group differences at POST-tests were analysed to investigate if the differences observed at PRE-tests were still present (data not shown). The differences in body composition observed at the PRE-tests between the 1^st^- and 2^nd^-year group, and the difference in BMD of L1–L4 between the PA-group and the CON-group (Table 2), were not observed at the POST-tests. The only statistically significant finding from this analysis was a significant 8.9% lower HR at 3.2 km/h in the PA-compared to the CON-group (Figure 2).

### 3.6. Coefficients of Variation

The coefficients of variation (CVs) for selected outcomes were computed. For fat mass, the CVs for the PA-group were 153 and 155%, in the 1^st^-year group the CVs were 92 and 95%, and in the CON-group the CVs were 75 and 69% (PRE and POST, respectively). The CVs for whole body BMD were 30 and 29% in the PA-group, and 15 and 14% in the CON-group (PRE and POST, respectively). With regards to the cardiovascular fitness measurements, we have reported the CVs of the HR during the last 30 seconds of the 4.8 km/h intensity, as they were very similar. In the PA-group, the CVs were 39 and 41%, in the 1^st^-year group the CVs were 24 and 25%, and in the CON-group the CVs were 22 and 20% (PRE and POST, respectively).

## 4. Discussion

The results of the present study indicates that adults with ID by participation in PA in a real-life setting for 14 weeks can improve their body composition, bone health and cardiovascular fitness. The amount of PA performed by the PA-group is above the weekly 300 minutes of PA, recommended for adults by the World Health Organization [22].

### 4.1. Body Composition

The PA-group lost 1.02 kg of fat mass and the 1^st^-year group lost 1.7 kg of fat mass, with no changes observed in body weight. The change in the total PA-group seems to be driven by the 1^st^-year group, as no changes were found for the 2^nd^-year group. The results of previous studies on the effect of physical activity on body composition for adults with ID are equivocal. Some studies on individuals with ID have reported reductions in fat mass of similar magnitude as the present study [12, 23] while other studies did not [10, 11]. In a meta-analysis of 6 RCT's assessing the effects of PA interventions on body composition in young adults with ID, Harris and colleagues reported no overall effect of physical activity interventions on body composition [24]. The studies included in the meta-analysis used interventions of 80–195 minutes of activity/week [24]. As Harris and colleagues suggested, this might be too little to elicit changes in body composition. In the present study, the participants in the PA-group performed ∼400 minutes of PA/week, of variable intensity. Thus, the training load imposed on our participants, should be enough to elicit the adaptations in body composition observed in the present study. This is supported by the findings from a systematic review on the effects of exercise on fat mass in the general population, where ∼10 weeks of either moderate- or high-intensity exercise, induced ∼2 kg fat mass reduction [25]. Food intake and the individual diets of the participants is another important variable to consider when studying changes in body composition [26]. Studies on individuals with ID have successfully improved body composition through multi-component interventions with exercise, diet, and motivation [27, 28]. Generally, the population have been shown to have a poor diet with a high energy intake from sugar and saturated fats [29] and a low intake of fruit and dietary fiber [30]. The diet of the participants was not monitored in the present study, so we are not able to gauge the effects of diet on the body composition or compare the diet of the participants to earlier studies. However, the SSADD employ a healthy approach regarding the food the participants are served while at the school. Thus, the results of the present study indicate that it is possible to reduce fat mass in the population of adults with ID, through increased amount of PA and a healthy lunch. Significant parts of the fat mass reduction seen in the present study could be located to the gynoid and android areas (Table 3). The android fat mass reduction for the 1^st^-year group is of significance in a health perspective, as the amount of android fat is a risk factor in relation to lifestyle diseases such as type II diabetes mellitus and cardiovascular diseases [31, 32]. Thus, our results points towards a positive effect of 14 weeks of PA performed at SSADD on fat mass.

### 4.2. Bone Health

The between group difference observed in whole body BMD and the within-group increase in right femur neck BMD in the PA-group, indicate that the PA-group benefitted from 14 weeks of PA, compared to the CON-group (Table 3). In addition to this, the higher BMD of L1–L4 in the CON-group compared to the PA observed at baseline measurements (Table 2), were not present at the POST-tests.

To our knowledge, the present study is one of the first to investigate the bone health in individuals with ID after a PA intervention, and the first to do so in adults. A previous study on 7–10 year-old boys with ID found an increase in femoral neck BMD after 6 months of exercise (165 min/week) [33]. Another study observed an increase in bone mineral content of the femur in a group of participants with Down syndrome between 10 and 19 years, after 21 weeks of exercise (50–60 min/week) [13]. Thus, the present study adds data to field bone health in the population with ID, with results indicating that PA has an osteogenic effect on individuals with ID. In the present study, the osteogenic effect of PA is evident as an increase in right femur neck BMD in the PA-group. There are differences between the present study and earlier research regarding bone adaptations to exercise that are important to consider when comparing the studies. First, it is common in this line of research to include activities aimed at inducing an osteogenic response i.e., activities which imposes strain and impact to the bones [34]. The study by Gonzalez-Agüero and colleagues used plyometric training and the study by Hemayatta's lab used running, hopping, and skipping exercises. In the present study, we did not monitor what type of activities were performed, and the accelerometers used were not able to distinguish between activities at this level. Additionally, the present intervention had a duration of 14 weeks, which is a short time to observe substantial adaptations in bone structure as a result of training [35]. These differences between the studies could be an explanation to why only increases in BMD in the right femur neck in the PA-group are observed. If the study period had been longer and had included activities specifically aimed at imposing strain and impact to the bones, the osteogenic effect could have been larger. However, the strength of the present results is the indication of an osteogenic effect of participation a generalized PA program for only 14 weeks. Finally, it should be noted that the previous studies investigated children and adolescents with ID, while this study was on adults. This difference in age groups is important to consider as BMD changes throughout development [36]. Currently there is no longitudinal research on the age related changes in BMD from populations with ID. With regards to the clinical relevance of the results, the increase of 0.017 g/cm^2^ of the right femur neck is within the range found in a systematic review of exercise studies on adults from the general population [37], and it may be of significance to the fracture risk of the participants [38].

At PRE-tests, the bone health of the participants in the present study was in alignment with previous research, regarding the BMD of the whole body [39], and of the lumbar vertebrae [40]. Our study population had higher *T*-scores and healthier distribution of *T*-scores of the right femur neck, than what was observed in earlier cross-sectional studies on similar populations [4, 41]. The lowest average *T*-score of the right femur neck in the present study was seen in the 1^st^-year group (−0.40), which was higher than the value reported by Zylstra and colleagues (−1.51) [4]. Zylstra and colleagues reported an osteoporosis prevalence (*T* ≤ −2.5) in the femur region of 22% and an osteopenia prevalence (−2.5 > *T* ≤ −1) of 33% in 36 participants with ID aged 19–29 years. The prevalence distribution of all participants in the present study was combined for comparison with the data reported by Zylstra and colleagues. We observed an osteoporosis prevalence of 0% and an osteopenia prevalence of 25.4%. Zylstra and colleagues did not report which model of DXA-scanner they used in their study, which could influence the results. In summary, adults with ID are a population with low *T*-scores of the femur and lumbar regions, but the population in the present study are not in as high risk of developing osteoporosis, as earlier studies have reported [4, 41].

### 4.3. Cardiovascular Fitness

The PA-group reduced their heart rate during the last 30 seconds on both speeds during the treadmill test (Figure 3), but we did not find any changes in the amount of O_2_ consumed per kg body weight during the submaximal exercise transitions on the treadmill test. We had expected to observe changes in O_2_ kinetics, as adaptations in this domain have been found to occur within two training sessions of either high-intensity training or low intensity endurance training in the general population [42].The reductions in HR point towards an improved cardiovascular function; the participants' hearts were able to meet the energy demand of the working muscles during the walking test with less beats per minute, possibly due to an increase in stroke volume [43]. The increase in cardiovascular fitness observed in the present study is in line with what was reported by earlier studies with similar target groups and intervention durations. Barwick and colleagues reported a decrease in HR in a after a 10-week functional training program [44], and studies employing combined training programs observed increases in peak O_2_ uptake velocity (VO_2_ peak) post intervention [10, 11]. It should be noted that some of the diagnoses included in the groups of the present study is associated with autonomic dysfunction, namely Down syndrome, and cerebral palsy [45, 46]. This could lead to an altered response of the HR than what is seen in the general population, however, our results are systematic with regards to the HR reduction during physical activity; it is reduced in the PA-group and are even more pronounced in the 1^st^-year group.

The set-up of the present study complicates the comparison with the mentioned traditional training studies, especially with regards to the physical load imposed on the participants. In the present study, we investigated the amount of PA performed by the PA-group while at SSADD and in their leisure (Figure 2), while traditional training studies delivered and monitored the PA conducted by the participants. The knowledge obtained from these earlier studies about the effect of PA on cardiovascular fitness, together with the results of the present study, suggests a link between the PA performed at SSADD and the cardiovascular adaptations observed. Measures of cardiac function such as reduced resting HR and improved HR recovery reduces all-cause mortality from cardiovascular diseases in the general population [47]. We did not obtain these measures in the present study, but a reduced HR at the same absolute intensities could point towards an improved health-enhancing cardiac function.

### 4.4. Limitations

The quasi-randomized design of the present study poses a selection bias. Individuals who choose to attend SSADD could have an overall higher interest in PA, than the general population with ID. We tried to meet this challenge by including individuals in our control group, who were recreationally physically active in their leisure. With regards to this, it would have been preferable to conduct accelerometry in the CON-group too, but this was not possible due to logistical challenges. This would have made direct comparisons on the activity level between the groups possible.

Our analysis of the coefficients of variation (CVs) of the fat mass data revealed CVs between 92 and 155% in the 1^st^-year and PA groups. Thus, the standard deviations of the fat mass measures were equal to or 1.5 times higher than the means. This is an indication of a wide distribution of the data around the mean in this measure and should be considered when interpreting this data. The CVs in the HR and BMD measures are all below 50%, which indicates a narrower distribution of the data around the mean in these measures.

## 5. Conclusion and Perspective

The present study found positive health effects in the domains of body composition, bone health and cardiovascular fitness in adults with ID after 14 weeks of PA. The PA performed at SSADD seems most efficient in improving the health the of 1^st^-year group. For participants who have spent one or more years at SSADD, the PA performed is likely to maintain their health in the parameters investigated in the present study. Future studies should employ a progressive increase in the intensity of the PA to investigate if this leads to health benefits for the participants in the 2^nd^ year-group. Improvements in the health parameters investigated in the present study could reduce the groups' risk of developing lifestyle diseases [14, 15, 48]. The researchers did not supervise the activities performed by the participants in the PA-group. Instead, this study focused on measuring the effects of an already existing PA option for the target group. Other studies have employed a similar approach, with regards to investigating well-established programs with PA for individuals with ID and have found health-enhancing effects as well [49, 50]. This approach is valuable in the research regarding how to improve the health status of individuals with ID.

## Figures and Tables

**Table 1:** Demographic data of participants from their first measurement.

Variables	PA	1^st^	2^nd^	CON
Demographic data				
Number of participants	52	32	20	14
Gender (f/m)	28/24	17/15	11/9	5/9
Age (years)	26.7 ± 1.2	26.7 ± 1.5	26.7 ± 1.4	29.1 ± 1.7
Height (cm)	163.3 ± 1.8	162.3 ± 2.2	165.1 ± 3.0	163.3 ± 2.4
Down syndrome	15	10	5	4
Cerebral palsy	6	3	3	1
Autism spectrum disorders	7	3	4	1
Other rare syndromes	5	2	3	2
Undifferentiated ID	19	14	5	6

This table provides an overview of the demographic data of the participants at the time they entered the study. The participants' age was recorded at PRE-tests The table includes the distribution of participants with different aetiologies of ID. The category “Other rare syndromes” include participants with Fragile X syndrome, William syndrome, tuberous sclerosis, Bardet Beidel syndrome, and Noonan syndrome. Abbreviations: f: female, m: male, cm: centimetres.

**Table 2:** Body composition and bone health measures at PRE-tests

Variables	PRE			
	PA	1^st^	2^nd^	Control
Body composition				
Body weight (kg)	73.90 ± 2.11	**75.98** **±** **3.46**^**b**^	72.42 ± 2.65	75.12 ± 5.72
Fat mass (kg)	25.74 ± 1.55	**29.25** **±** **2.45**^**bb**^	25.96 ± 3.87	25.74 ± 4.3
Fat free mass (kg)	44.14 ± 1.06	44.24 ± 1.62	44.08 ± 1.42	47.11 ± 1.96
Android fat (kg)	2.46 ± 0.18	**2.71** **±** **0.31**^**bb**^	2.27 ± 0.21	2.27 ± 0.46
Gynoid fat (kg)	4.70 ± 0.26	**4.96** **±** **0.40**^**b**^	4.52 ± 0.51	4.48 ± 0.70
BMI (kg/m^2^)	28.19 ± 0.88	**29.05** **±** **1.36**^**b**^	27.58 ± 1.16	28.05 ± 2.45
Whole body fat percent	36.72 ± 1.35	**38.12** **±** **2.14**^**bb**^	35.73 ± 1.74	32.74 ± 2.51
Bone health (BMD and *T*-scores)				
Whole body (g/cm^2^)	1.155 ± 0.014	1.163 ± 0.026	1.149 ± 0.015	1.208 ± 0.021
Whole body *T*-score	0.26 ± 0.126	0.40 ± 0.239	0.16 ± 0.136	0.47 ± 0.245
L1-L4 (g/cm^2^)	**1.140** **±** **0.016**^**a**^	1.129 ± 0.026	1.150 ± 0.020	1.217 ± 0.031
L1-L4 *T*-score	−0.53 ± 0.140	−0.56 ± 0.262	−0.51 ± 0.158	−0.43 ± 0.288
Left total femur (g/cm^2^)	0.981 ± 0.015	0.980 ± 0.028	0.981 ± 0.017	1.023 ± 0.028
Left total femur *T*-score	−0.44 ± 0.119	−0.43 ± 0.225	−0.45 ± 0.133	−0.27 ± 0.259
Right total femur (g/cm^2^)	0.981 ± 0.015	0.987 ± 0.025	0.976 ± 0.018	1.027 ± 0.028
Right total femur *T*-score	−0.46 ± 0.114	−0.40 ± 0.203	−0.50 ± 0.134	−0.24 ± 0.257
Left femur neck (g/cm^2^)	0.972 ± 0.015	0.960 ± 0.028	0.981 ± 0.018	1.007 ± 0.027
Left femur neck *T*-score	−0.36 ± 0.116	−0.46 ± 0.210	−0.30 ± 0.134	−0.24 ± 0.257
Right femur neck (g/cm^2^)	0.980 ± 0.015	0.971 ± 0.024	0.986 ± 0.019	0.995 ± 0.029
Right femur neck *T*-score	−0.32 ± 0.109	−0.40 ± 0.178	−0.26 ± 0.140	−0.33 ± 0.271

PRE-test values as raw means ± standard error of the mean (SEM). Abbreviations: L1- L4 : Lumbar vertebrae 1 to 4. Significance codes: ^**a**^: Difference compared to the Control group. ^**b**^: Difference compared to 2nd-year participants. Significance codes: “^aa^/^bb^” denotes *p* < 0.01, “^a^/^b^” denotes *p* < 0.05.

**Table 3:** Changes within and between groups over time in body composition and bone health.

Variables	Within group Δ-values from PRE to POST-tests				Between group change scores from PRE to POST-tests			
	ΔPA	Δ1^st^	Δ2^nd^	ΔCon	ΔPA—ΔCon	Δ1^st^—ΔCon	Δ2^nd^—ΔCon	Δ1^st^—Δ2^nd^
Body composition								
Body weight (kg)	−0.44 ± 0.52	−1.10 ± 0.72	0.01 ± 0.69	−0.31 ± 1.16	0.14 ± 1.25	−1.05 ± 1.31	0.06 ± 1.27	−1.11 ± 0.99
Fat mass (kg)	**−1.02** **±** **0.43**^*∗*^	**−1.71** **±** **0.59**^*∗*^	−0.53 ± 0.56	0.23 ± 0.94	−1.24 ± 1.02	−2.18 ± 1.07	−1.00 ± 1.04	−1.18 ± 0.81
Fat free mass (kg)	0.29 ± 0.17	0.22 ± 0.25	0.31 ± 0.24	0.24 ± 0.38	0.04 ± 0.41	−0.06 ± 0.45	0.04 ± 0.44	−0.09 ± 0.35
Android fat (kg)	−0.08 ± 0.06	**−0.20** **±** **0.08**^*∗*^	0.01 ± 0.07	0.04 ± 0.13	−0.12 ± 0.14	−0.028 ± 0.14	−0.07 ± 0.14	−0.21 ± 0.11
Gynoid fat (kg)	**−0.27** **±** **0.08**^*∗*^	**−0.35** **±** **0.11**^*∗*^	−0.23 ± 0.11	−0.06 ± 0.18	−0.22 ± 0.20	−0.32 ± 0.21	−0.21 ± 0.20	−0.11 ± 0.16
BMI (kg/m^2^)	−0.18 ± 0.19	−0.40 ± 0.26	0.00 ± 0.25	0.07 ± 0.42	−0.11 ± 0.45	−0.43 ± 0.48	−0.01 ± 0.47	−0.42 ± 0.36
Whole body fat percent	**−0.97** **±** **0.32**^*∗∗*^	**−1.46** **±** **0.45**^*∗∗*^	−0.62 ± 0.43	−0.16 ± 0.72	−0.81 ± 0.78	−1.47 ± 0.82	−0.63 ± 0.80	−0.84 ± 0.63
Bone health (BMD)								
Whole body (g/cm^2^)	0.008 ± 0.004.	0.004 ± 0.006	0.010 ± 0.005	−0.015 ± 0.008	**0.024** **±** **0.009**^*∗*^	0.019 ± 0.010	**0.025** **±** **0.010**^*∗*^	−0.006 ± 0.008
L1–L4 (g/cm^2^)	−0.001 ± 0.005	0.005 ± 0.007	−0.006 ± 0.006	−0.020 ± 0.010	0.019 ± 0.011	0.026 ± 0.012	0.015 ± 0.012	0.011 ± 0.009
Left total femur (g/cm^2^)	0.002 ± 0.003	0.001 ± 0.005	0.003 ± 0.005	−0.007 ± 0.007	0.009 ± 0.008	0.008 ± 0.009	0.010 ± 0.009	−0.002 ± 0.007
Right total femur (g/cm^2^)	0.007 ± 0.004	0.003 ± 0.006	0.011 ± 0.006	−0.001 ± 0.009	0.008 ± 0.010	0.003 ± 0.011	0.012 ± 0.011	−0.009 ± 0.008
Left femur neck (g/cm^2^)	0.011 ± 0.006	0.006 ± 0.009	0.008 ± 0.013	−0.008 ± 0.013	0.003 ± 0.141	−0.002 ± 0.016	0.007 ± 0.015	−0.009 ± 0.012
Right femur neck (g/cm^2^)	**0.017** **±** **0.007**^*∗*^	0.017 ± 0.010	0.017 ± 0.009	0.007 ± 0.015	0.010 ± 0.016	0.012 ± 0.0178	0.012 ± 0.017	0.000 ± 0.013

Changes within the groups are reported as delta values from PRE to POST-tests (POST—PRE) ± standard error of estimate. Differences between groups are reported as change scores (i.e., the differences between the delta-values of the groups) ± standard error of estimate. Abbreviations: BMI: Body mass index, BMD: Bone mineral density, L1–L4: Lumbar vertebrae 1 to 4. Significance codes: “^*∗∗*^” denotes *p* < 0.01, “^*∗*^” denotes denotes *p* < 0.05, “.” denotes *p* < 0.1.

## Data Availability

The data used to support the findings of this study are available from the corresponding author upon request.

## Supplementary Materials

Supplementary Materials Appendix 1 Participants, characteristics, and distribution on the different outcomes. Supplementary Materials.
